# Supplementary figures and images for: Measuring User Engagement with a Socially Connected, Gamified Health Promotion Mobile App
Source: Int J Environ Res Public Health. 2022 May 5;19(9):5626. doi: 10.3390/ijerph19095626 (PMC9102982; doi:10.3390/ijerph19095626)

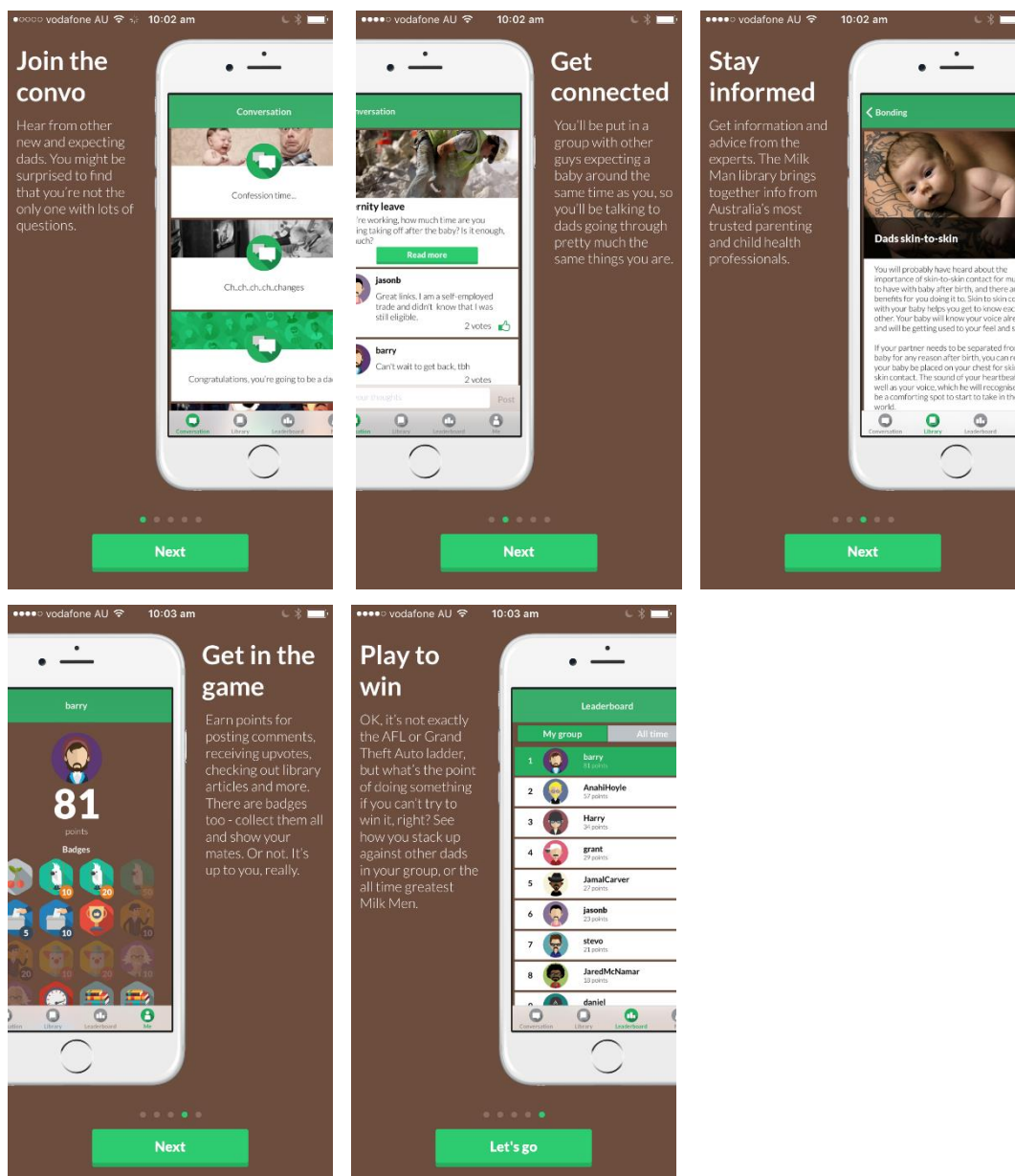

**Supplementary Figure S1: Milk Man onboarding screens**

Supplement: Supplementary file 1 [file ijerph-19-05626-s001.zip › ijerph-1687895-supplementary.pdf]
